# A late presentation of inguinoscrotal cutaneous squamous cell carcinoma (cSCC) masquerading as hidradenitis suppurativa—a case report

**DOI:** 10.1093/jscr/rjad459

**Published:** 2023-08-09

**Authors:** Elias Edward Lahham, Salem Billan, Fadi Atrash, Walid Fararja

**Affiliations:** Department of Radiation Oncology, Augusta Victoria Hospital, Palestinian Authority, Palestine; Head and Neck Unit, Joseph Fishman Oncology Center, Rambam Health Care Campus, Haifa, Israel; Department of Radiation Oncology, Augusta Victoria Hospital, Palestinian Authority, Palestine; Department of Radiation Oncology, Augusta Victoria Hospital, Palestinian Authority, Palestine

**Keywords:** Hidradenitis suppurativa, Squamous cell carcinoma, PD1 inhibitors

## Abstract

Hidradenitis suppurativa (HS) is a chronic inflammatory skin condition that affects apocrine gland-bearing skin in intertriginous areas; it is characterized by persistent or recurrent abscesses that culminate in a purulent discharge, sinuses and scarring. Although it is a common condition, it is rarely associated with cutaneous squamous cell carcinoma (cSCC). Whereas the female-to-male ratio of HS is 3:1, SCC in HS has a female-to-male ratio of 1:5. In this study, we present a 50-year-old male patient with a late presentation of inguinoscrotal cSCC with extensive ulceration and erythema that was hidden by neglected HS and the presence of underlying human papillomavirus in the affected area. The histomorphologic and immunohistochemical findings support the diagnosis of cSCC. Timely detection is the key to improve prognosis, regular clinical examination and biopsy of any suspicious lesions in high-risk patients is highly recommended.

## INTRODUCTION

Hidradenitis suppurativa (HS) is a chronic, relapsing and remitting inflammatory disease of the skin with high morbidity rates and an overall disease prevalence of 1% [1]. The development of squamous cell carcinoma (SCC) is 4.6 times more likely in patients with HS than in the general population. Although HS is more common in women, its complication and malignant transformation are predominantly in men [2]. Cutaneous SCC (cSCC) arising in HS is an uncommon consequence that may occur due to chronic inflammation or the presence of underlying human papillomavirus (HPV) in affected areas that lead to epidermal hyperproliferation [3, 4]. cSCC tumors that arise from chronic inflammatory processes, called de novo cSCC, are higher aggressive than conventional cSCC [3, 4]. In our patient, HPV seems to have played a crucial oncogenic role, explaining the aggressive progression of HS to cSCC [3]. This study aims to alert physicians to reduce the threshold for taking biopsies from wounds that are chronic or have changes in characteristics and to shed light on the new era of biologic therapies in cSCC treatment.

## CASE PRESENTATION

**Figure 1:**
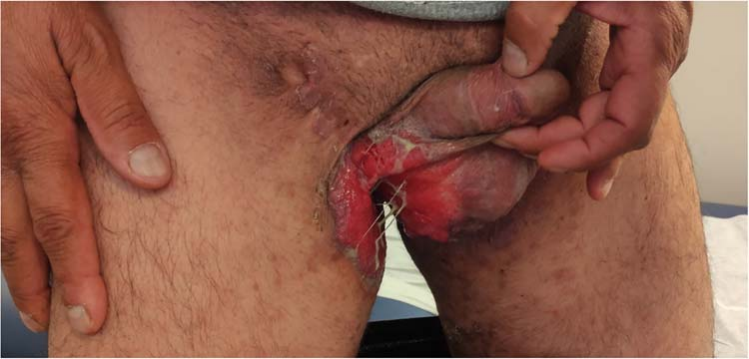
Ulcerated cutaneous squamous cell carcinoma arising in HS in the right groin with scrotal involvement.

**Figure 2:**
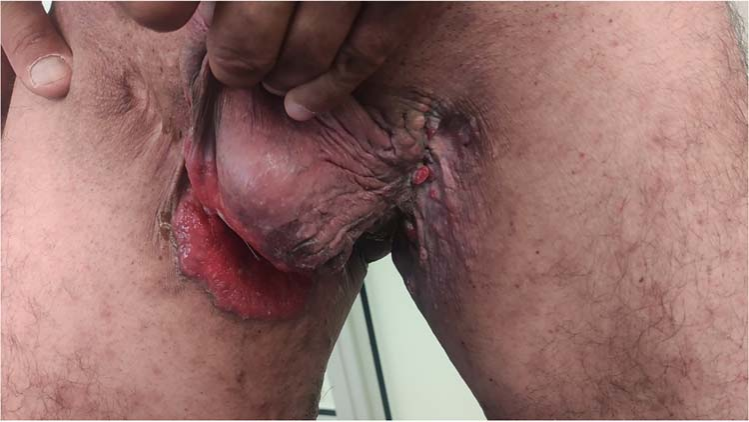
Small nodules and papules in the left groin, largest (0.8^*^0.5 cm), with skin pigmentation and extensive old surgical scars. Histopathological assessment of the nodules confirms the diagnosis of cSCC.

A 50-year-old male patient married in a monogamous relationship, heavy smoker, known case of diabetes mellitus type 2, recurrent chronic groin HS since 5 years ago and genital verrucous papules that were diagnosed and treated 20 years ago. Presented to our clinic complaining of itchy, erosion redness and yellowish discharge in the groin area with nodules on the medial side of his thighs bilateral for 2 years duration, which increased in severity in the last 6 months, during this period the patient poorly followed up due to embarrassing issue, he was initially treated with recurrent surgical incision/ drainage and antibiotics as he is a known case of chronic hidradenitis supporitiva. On arrival, He was hemodynamically stable and afebrile. Physical examination showed extensive skin ulceration 8^*^10 cm on the left inguino-scrotal side with purulence discharge (Fig. 1), a multiple pink nodules in the groin area with dark pigmentations and extensive old surgical scars was noted (Fig. 2). His previous histology reports 20 years ago from genital verrucous papules revealed epithelial papillary overgrowth with no sign of malignancy, subsequent excisional biopsies consist of chronically inflamed condyloma accumulation. Due to the chronicity of the patient’s complain, a new skin biopsy was ordered, the Immunohistochemical studies of the tumor cell is positive for cytokeratin CK7 and CK5/6, they are focally positive for P63, while negative for CK18, CK20, synaptophysin, chramogramin, S100 and HMB45, support the diagnosis of cSCC. The polymerase chain reaction (PCR) was positive for high-risk HPV 16. Computerized tomography scan done showed localized disease with no distant metastasis. Ultrasound-guided biopsy of the inguinal lymph node was normal. Interdisciplinary team discussion concluded that this case represents a cSCC of the genitalia due to chronic wound inflammation with underlying viral etiology, which is considered non-operable and should respond to anti programmed cell death (PD-1) therapy that is the first choice of treatment. The patient was referred to the oncology ward for starting immunotherapy. The patient tolerated well the pembrolizumab with no significant adverse events and was advised to visit the dermatologist regularly for proper skin examinations. If there is an insufficient response to the immunotherapy or recurrent disease, radiotherapy could be one of the treatment plans, finally as HPV is the commonest sexually transmitted disease, his wife advised to do a gynecological examination with a pap smear for early detection of cellular changes on the cervix that might become cervical cancer if left untreated.

## DISCUSSION

HS is a chronic painful skin condition that causes recurrent skin abscesses and scarring. SCC is a late and rare complication of HS, with malignant transformation occurring in 3.2% of patients with HS [5]. Although its exact pathogenesis is still unknown. The main causes of the development of SCC in HS appear to be epidermal hyperproliferation in long-standing inflammation and local spread of malignant cells through the extensive sinus tracts [2]. Although HS mostly affects females, males with HS lesions appear to be at greater risk of developing cSCC [2]. In a review of 52 cases with SCC lesions arising in HS, results found that 57% of patients died within 2 years [6]. The high mortality may be due to a combination of late recognition, ineffective medical management and aggressive SCC arising from chronic wounds [6]. Moreover, tobacco use and HPV infection may be associated with the malignant transformation of chronic HS into SCC [7]. Lavogiez *et al*. [6] found that the presence of HPV combined with chronic inflammation and defective skin cellular immunity can potentiate the malignant degeneration in HS. Thereby, the critical oncogenic role of HPV as local cofactors may explain why the majority of cSCC in HS occurs. Huang *et al.* [8] showed a ~27-year latency period from the diagnosis of HS to the development of cSCC. In our patient, the malignant transformation occurred after ~5 years of evolution, suggesting that the presence of HPV combined with chronic inflammation of HS are synergistic effects in the promotion of cSCC. The majority of cSCC cases were treated successfully with standard wide local excision and/or radiotherapy, However, 5% of cSCC were considered unrespectable either locally advanced or metastatic disease, which have a dismal outcome. Fortunately, novel immunotherapy that target the PD-1 pathway is causing a paradigm shift in cSCC with better tolerability and fewer side effects than traditional modalities [9–11], which recently considered as a suitable treatment to reduce the morbidity and mortality of cSCC developing in HS [12, 13]. In our case, Anti PD-1 therapy was started as a first-line treatment due to the ineligibility of the patient to surgery.

## CONCLUSION

SCC arising in the setting of HS is very rare, chronic inflammation and repetitive injuries may increase the risk of developing cSCC at the HS site, additionally, the HPV background has been implicated in the promotion of malignant transformation. Furthermore, the role of HPV vaccination in the prevention of cSCC, and possibly its treatment, in HS patients is an interesting topic for future studies.

## Data Availability

The data used to support the findings of this study are included within the article.
